# Dialogue through film: engaging midwives, TBAs, and mothers to improve maternal health outcomes in Ghana’s Volta region

**DOI:** 10.3389/fgwh.2025.1507547

**Published:** 2025-11-25

**Authors:** Sandrina Koppitz, Obehi Oseyomon Iziduh, Sonja Liggett-Igelmund, Janet Michel

**Affiliations:** 1 Moving Frame e.V., Berlin, Germany; 2 MPH, Accra, Ghana; 3 Meeting Bismarck Gododo Ghana Geburts- und Kinderhilfe e.V., Köln, Germany; 4 Global Health Mentorship, Copenhagen, Denmark

**Keywords:** maternal mortality rate, maternal care, maternal mortality, midwives, traditional birth attendants, rural Ghana, maternal health, pregnancy

## Abstract

**Background:**

Maternal mortality in Ghana remains high at 263 deaths per 100,000 live births, with the Volta Region showing particularly concerning figures; 37.2% of women give birth without a skilled provider. Many rely on Traditional Birth Attendants (TBAs), whose role remains unofficial and unregulated. Causes of maternal death include hemorrhage, sepsis, hypertensive disorders, and unsafe abortions, compounded by limited access to emergency care and mistrust in clinical settings. To create a dialogue between the stakeholders to reduce maternal mortality, we want to screen the documentary *Among Us Women.* The documentary is set in rural Ethiopia, explores the choice between home and hospital birth. A first screening in Ghana revealed similar challenges—highlighting women's trust in TBAs and dissatisfaction with clinical care. The film successfully opened dialogue between midwives and TBAs, showing potential for collaboration.

**Hypothesis:**

We propose that film screenings followed by inclusive dialogue will improve mutual respect and cooperation between TBAs and clinical staff in rural areas, leading to earlier interventions and reduced maternal deaths within one year.

**Method:**

A shortened, Ewe-dubbed version of the film will be shown in 60 randomly selected communities in the Volta Region, reaching 500+ stakeholders. Roundtables will follow each screening, supported by trained facilitators. Discussions and follow-up evaluations will inform a “community needs catalog” and provide data for potential national scale-up.

**Discussion:**

The main question is if setting up an efficient dialogue as a result of a film screening in other communities is replicable. To set up a reliable study, test screenings of the film upfront will refine the approach, addressing language, group size, and ethical concerns. Using relatable storytelling and dialogue, the project fosters empathy, shared responsibility, and cultural sensitivity; laying the groundwork for long-term improvements in maternal health.

## Introduction

## Background

### Current state of maternal health in the Volta region, Ghana

Maternal mortality remains a critical public health challenge in Ghana, with a maternal mortality ratio (MMR) of 263 deaths per 100,000 live births (1). This issue is central to Sustainable Development Goal 3, which aims to ensure healthy lives and promote well-being for all.

A maternal death is defined as the death of a woman from any cause related to or aggravated by pregnancy or its management—excluding accidental or incidental causes—during pregnancy, childbirth, or within 42 days of termination of pregnancy, regardless of the duration or site of the pregnancy (2).

A study conducted between 2004 and 2008 at the Korle Bu Teaching Hospital, led by Dr. Edmund M. Der, found that 12.1% of all deaths among women of reproductive age (15–49 years) were pregnancy-related. Of these, 81.5% occurred either in the community or within 24 h of admission to a health facility (3). The majority (70%–80%) of maternal deaths were due to direct obstetric causes—primarily hemorrhage, hypertensive disorders (including intracranial hemorrhage, cardiac failure, and hypertensive encephalopathy), genital tract sepsis, and early pregnancy complications. Unsafe abortions also remain a significant contributor (3–5). Women aged 25–29 are the most affected, accounting for 25.9% of maternal deaths, while the risk significantly decreases with age (3).

Beyond medical causes, maternal mortality in Ghana is exacerbated by social, economic, and cultural factors. Only about half of women live within a two-hour journey (via motorized transport) to the nearest emergency obstetric and neonatal care facility (6). For many, the cost of transport is prohibitive, resulting in delays or the need to travel on foot, which is further increasing risk.

In rural areas, 31.1% of women—and as many as 37.2% in the Volta Region—give birth without the assistance of a skilled health provider (7). Traditional Birth Attendants (TBAs) continue to play a central role in maternal care in remote communities. TBAs typically support pregnant women throughout their pregnancy, providing emotional and cultural guidance. However, their role is not officially recognized under Ghanaian law, and collaboration between TBAs and licensed midwives is limited, especially as TBAs are legally restricted from practicing in areas with a registered midwife.

### Background of the film *Among US Women* and the associated impact campaign

The German-Ethiopian documentary *Among Us Women* (8) by Sarah Noa Bozenhardt (Evolution Film) explores the topic of giving birth at home or with a medical skilled institution from the empathetic perspectives of all stakeholders—clinical midwives, TBAs, and expecting mothers. Although set in rural Ethiopia, the film's context and themes closely mirror realities in rural Ghana.

The protagonist Huluager faces a choice between delivering her fourth child at a hospital or at home with a TBA. Life-threatening complications during childbirth at home are ultimately resolved through timely medical intervention, saving her life. The film captures the complex dynamics of choice, trust, and survival surrounding childbirth.

One key insight from the film's production was the realisation that, in relationships marked by power imbalances (especially class and education), the less empowered party often conceals their actions—despite knowing what might be expected of them.

Following its premiere at DOK Leipzig, the film sparked significant interest, leading to the development of an impact campaign. Ethiopian and German teams partnered with midwifery associations, NGOs, and educational institutions. The film has since been incorporated into midwifery training programs and university courses on global health. While community screenings in Ethiopia were initially planned, they were halted due to the civil war.

As an alternative, the NGO *Meeting Bismarck e.V.* proposed piloting the intervention in Ghana's Volta Region, starting with screenings for midwives, TBAs, and (expectant) mothers at the Have Health Centre.

### Initial observations from Ghana screenings

The first screening revealed the project's transformative potential. A 90-minute discussion followed, highlighting the disconnect between expectant mothers and clinical care systems. Many mothers felt dismissed or disrespected in clinical settings, resulting in mistrust and a preference for TBAs.

Examples included:

- A woman being left exposed, naked and with wide-spread legs, without privacy.
- Others recounting midwives yelling at them during labor.
- One mother credited her child's survival to a TBA, after a midwife had predicted the child's death.

These stories underscored the critical need for empathy and dignity in care. There was consensus that combining TBAs’ emotional support with the clinical expertise of midwives could improve maternal outcomes.

Surprisingly, midwives at the Have Health Centre had never met TBAs from their own district in Afadjato South. The screening became a catalyst for dialogue, resulting in a spontaneous knowledge exchange and training. TBAs, including retired farmers and pastors, expressed interest in collaboration. Midwives welcomed the idea as well.

Discussing the experience of the screening with the maternal health department Ghana Health Service (GHS) in the Volta Region, they recognised this as an opportunity to investigate why many women continue to choose home births, despite well-documented medical risks.

To generate concrete data for decision-makers within the Ghana Health Service; and to develop a potential blueprint for replication in other regions, we designed a study that uses the documentary film as a catalyst, followed by inclusive round-table discussions with all key stakeholders on equal footing.

## Hypothesis

The screening of the documentary *Among Us Women* will serve as a starting point for meaningful discussions on maternal health. By creating inclusive platforms for dialogue, bringing together clinical midwives, Traditional Birth Attendants (TBAs), and (expectant) mothers, we aim to foster mutual understanding, respect, and collaboration.

We hypothesize that in communities where such partnerships are established, maternal mortality will decrease within one year. This anticipated improvement is due to the earlier identification of high-risk pregnancies and more timely referrals to appropriate healthcare services.

A collaborative model between clinical midwives and TBAs in the Volta Region holds significant potential. Midwives contribute essential medical expertise, while TBAs provide emotional support, cultural continuity, and the trust of their communities.

Evidence shows that many maternal deaths are linked to limited awareness of obstetric danger signs. This was stated as well in “Maternal Death in Rural Ghana: A Case Study in the Upper East Region of Ghana” (9). Engaging and training TBAs, who are often the first point of contact for pregnant women in rural areas, can help close this knowledge gap. Seeing symptoms on time enables earlier intervention, especially by encouraging mothers to seek prompt emergency treatment or even allows the TBA to contact midwives for first aid instructions. This will improve outcomes for both mothers and newborns.

## Method

In collaboration with the GHS, we will screen a shortened version of *Among Us Women* in 60 randomly selected communities across the Volta Region. The film will be dubbed in Ewe to ensure accessibility. The target audience includes TBAs, midwives, community health nurses, Queen Mothers, and (expectant) mothers who have delivered at home or in health facilities. In total, we aim to reach at least 500 stakeholders.

Following each screening, moderated roundtable discussions will explore themes raised in the film. Facilitators trained in non-violent communication will guide conversations in a welcoming, respectful environment. The goal is to use Huluager's story to draw out the experiences of the women in a non-threatening way.

Each discussion will last around one hour, with a limited number of guided questions to stimulate open dialogue. We aim to gain a deeper understanding of the motivations and needs of women and to identify what improvements are necessary in the work of both TBAs and midwives to ensure better childbirth experiences.

Four researchers (two local, two international) will observe the screenings and discussions, conducting pre- and post-screening interviews to assess current practices, inter-group relationships, and knowledge exchange. The participants/ the audience will be informed before the screening that they are part of a study, and as for their consent.

Additionally, five screenings will be held at emergency obstetric care centers like Ho Teaching Hospital, involving professional staff, trainees, and invited community members (e.g., respected TBAs and mothers with unique stories). These sessions aim to explore the perspectives of highly trained clinical professionals while opening a space to reflect on the insights and experiences of non-clinical maternal care providers outside of the formal work environment.

A key outcome will be a collaboratively created “community needs catalog,” summarizing stakeholder-identified priorities. This catalog will be shared with communities and health authorities.

Over the following year, community health nurses will monitor collaborations between TBAs and midwives. After 12 months, communities will be revisited for follow-up questionnaires and discussions to evaluate the project's impact. Researchers will correlate these findings with MMR data and geographic proximity to health facilities.

The results will be presented to regional and national health authorities as a potential blueprint for scale-up.

## Discussion

The main goal is to replicate efficient discussion with all stakeholders on an eye level to gain deeper insight into the needs of the women. Before the main study begins, we will record the Ewe-language audio version of the film and develop a comprehensive questionnaire. Five test screenings will be conducted to assess:

- Comprehension across Ewe dialects
- Impact of international researcher presence
- Influence of different host institutions (local NGOs, GHS facilities, traditional village assemblies)
- Methods to ensure participant anonymity (considering literacy barriers)
- Optimal group size for productive discussion
- Impact of gender, professional authority, or power dynamics in attendance
- Balancing community selection by size, infrastructure, and access to health services

The study will run in close collaboration with the GHS, who will present the findings to the Ministry of Health. If successful, the results may support official recognition and training of TBAs in line with WHO's revised position acknowledging the role of trained TBAs in reducing maternal mortality (10).

## Sustainability

Education and behavior change are fundamental to sustainable health outcomes. Through the relatable story of Huluager, the project fosters empathy, awareness, and personal reflection among participants.

These conversations are likely to leave a lasting impression, particularly on healthcare providers, about the importance of mental well-being, dignity, and respectful care.

By facilitating open dialogue between the traditional and biomedical sectors, the project fosters mutual understanding and collaboration, helping women make informed, empowered decisions about childbirth.

The project aims to demonstrate how media and community-based dialogue can meaningfully contribute to maternal health, laying the foundation for long-term change.
